# Periplasmic flagella in *Borrelia burgdoferi* function to maintain cellular integrity upon external stress

**DOI:** 10.1371/journal.pone.0184648

**Published:** 2017-09-12

**Authors:** Bharath Kumar, Kelly Miller, Nyles W. Charon, Justin Legleiter

**Affiliations:** 1 The C. Eugene Bennett Department of Chemistry, West Virginia University, Morgantown, West Virginia, United States of America; 2 Department of Microbiology, Immunology and Cell Biology, Robert C. Byrd Health Sciences Center, West Virginia University, Morgantown, West Virginia, United States of America; 3 WVU nanoSAFE, West Virginia University, Morgantown, West Virginia, United States of America; 4 Blanchette Rockefeller Neurosciences Institute, West Virginia University, Morgantown, West Virginia, United States of America; University of Kentucky College of Medicine, UNITED STATES

## Abstract

Tapping mode atomic force microscopy (AFM) in solution was used to analyze the role of the internally located periplasmic flagella (PFs) of the Lyme disease spirochete *Borrelia burgdorferi* in withstanding externally applied cellular stresses. By systematically imaging immobilized spirochetes with increasing tapping forces, we found that mutants that lack PFs are more readily compressed and damaged by the imaging process compared to wild-type cells. This finding suggest that the PFs, aside from being essential for motility and involved in cell shape, play a cytoskeletal role in dissipating energy and maintaining cellular integrity in the presence of external stress.

## Introduction

**Fig 1:**
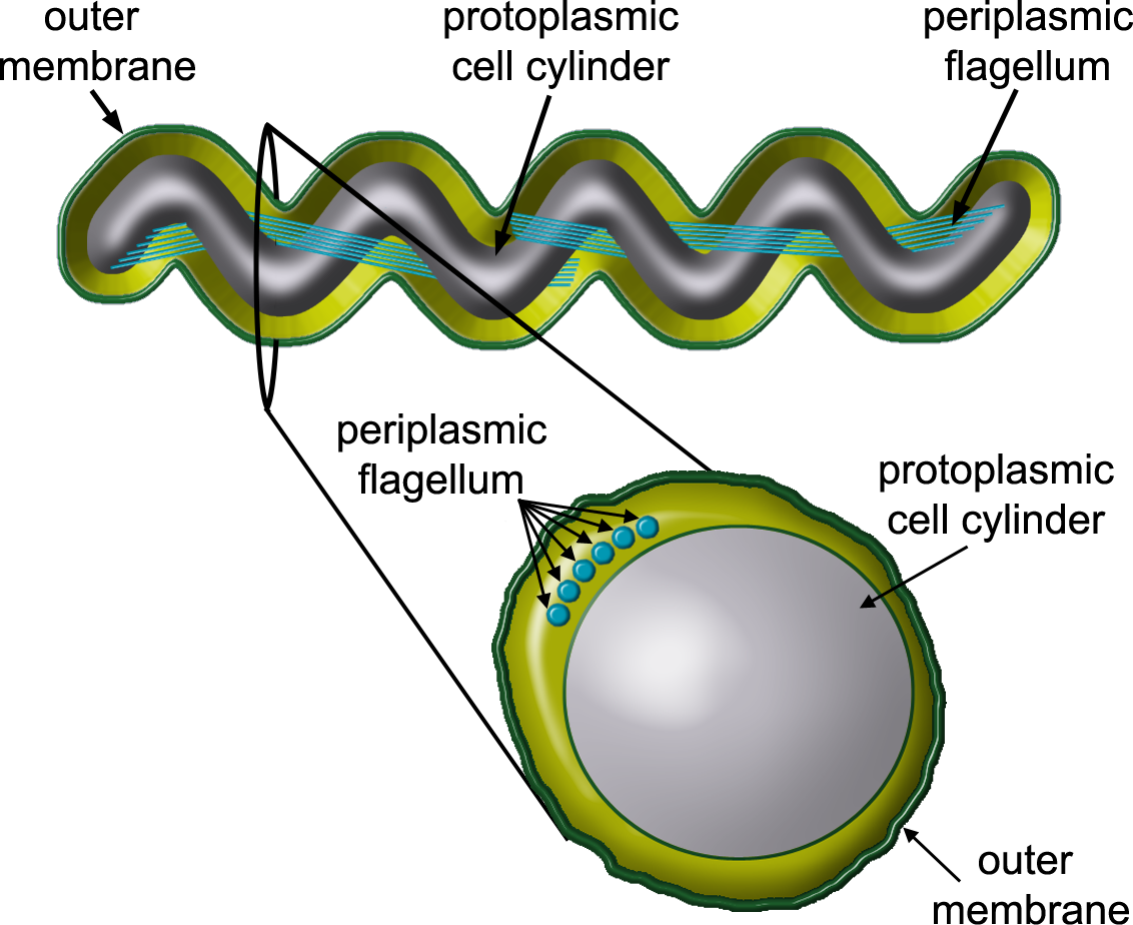
Schematic of *B*. *burgdorferi* structural organization. *B*. *burgdorferi* has a planar, flat-wave morphology that is the result of the periplasmic flagellum (PF) wrapping around the cell body between the protoplasmic cell cylinder and the outer membrane. The cross section demonstrates that the periplasmic flagella are ordered in a ribbon-like manner, wrapping around the cell body. The PFs overlap in the center of the cell. Schematic not drawn to scale.

Spirochetes comprise a unique phylum of motile bacteria distinguished by cell morphology, as their cell bodies have either a helical or flat-wave morphology depending on the species. With few exceptions, spirochetes consist of a cell cylinder and periplasmic flagella (PFs) that are contained within an outer membrane (Fig 1) [1–5]. The PFs are structurally similar to the flagella of other bacteria [6], as each consists of a basal body-motor complex, hook, and filament (see recent reviews on spirochete structure and motility [1,3,4,7–9]). In addition, the stiffness of the PFs of *B*. *burgdorferi* is similar to that of the flagella of other bacteria [10]. However, being located in the periplasmic space between the protoplasmic cell cylinder and outer membrane, the flagella of spirochetes are isolated from the surrounding environment. PFs function in cellular motility by the action of rotary motors located at their base. The flagellar hooks serve as universal joints linking the filaments to the motors. The hook structure of the spirochete hooks are unique, as the proteins that comprise the hook form high molecular weight complexes that are stabilized by covalent cross-linking [7,11–13].

Due to its role in Lyme disease and its similarity to the uncultivable spirochete *Treponema pallidum* which causes syphilis, considerable research effort has been focused on understanding the motility and morphology of the spirochete species *B*. *burgdorferi* [1,3,4,7,9]. This particular spirochete has a relatively long aspect ratio (10–20 μm long compared to ~ 0.3 μm thick) and exhibits a characteristic flat-wave morphology (Fig 1). There are typically 7–11 subterminally attached PFs at each cell end that form elegantly structured ribbons that tightly wrap clockwise around the cell body (Fig 1) [7,14–16]. The PFs within these ribbons are very close together, as they are spaced approximately 3.0 nm or less from each other. In cross-section, each ribbon occupies slightly less than one eighth of the cell circumference [14]. In addition, these ribbons are closely juxtaposed between the outer membrane sheath and cell cylinder such that the outer membrane bulges in their vicinity [14]. Motor rotation augments ribbon formation, as paralyzed *motB* mutants still form ribbons near the cell ends with the cell having a flat-wave in that region, but the central part of the cell lack both ribbon formation and the flat-wave morphology [15]. Recently, *flhB* motor mutants, which lack the unique structure of spirochete motors referred to as the collar, still synthesized flagella but failed to form the PF ribbon. Instead, the PFs were oriented in the opposite direction toward the proximal cell ends in this mutant, and were rod–shaped [17]. Thus, both PF rotation and motor mediated filament orientation are necessary for ribbon formation and the flat-wave morphology.

Genetic analysis of specific mutants has been critical in understanding both spirochete motility and chemotaxis, and their role in disease. Our laboratory has focused on the flagellar filament proteins FlaB and the hook protein FlgE of *B*. *burgdorferi*. Mutations in either of the encoding genes result in non-motile cells with a rod-shaped morphology [13,18,19]. The common motif among these mutants is that they lack the formation of flagellar filaments. Recent models of locomotion indicate that the cytoskeletal function of PFs are mechanistically important for motility in several spirochete species [1,3,4,7,20–27]. In general, motility is accomplished by backward propagating flat waves along the cell body that are produced by coordinated rotation of the PFs [1,3,4,7,9]. Most important, mounting genetic based evidence supports an intimate dependence of virulence on motility and chemotaxis [3,4,7,15,19,23,26,28,29].

While it is clear that PFs play a prominent role in determining the shape of *B*. *burgdorferi* and motility, a complete understanding of how spirochetes maintain their shape and cellular integrity in response to environmental and mechanical stresses is lacking. Here, we investigate the role PFs play in maintaining cellular integrity when an external, localized stress is applied repeatedly perpendicular to the extended axis of the cell. This is accomplished by using tapping mode atomic force microscopy (AFM) in solution to simultaneously deform and image immobilized wild-type or two distinct mutants of *B*. *burgdorferi*. The mutants used in this study were *flaB-* (unable to form the flagellar filament), and *flgE-* (unable to form the flagellar hook and filament) [12,13,18,30,31]. Whereas the motors of both mutants are intact [31], each mutant has completely impaired motility and has a rod-shaped morphology. The two different mutants were used to reinforce that the lack of PFs was the underlying basis of the observed differences compared to the wild-type. Our analysis indicates that the PFs of *B*. *burgdorferi* play a critical role in resisting cellular compression and preventing permanent damage associated with externally applied stresses.

## Materials and methods

### Bacterial strains, culture conditions, and cell preparation for AFM

High passage attenuated *B*. *burgdorferi* strain B31A and its specifically derived mutants were used throughout. These mutants encode a kanamycin resistance cassette (*aphI*) insertion mutation in major flagellin protein *flaB* (mutant MC-1), the flagellar hook gene *flgE* (mutant SC-E1), or the *cheW2* gene (deletion mutant Δ*W2*) [13,18,32]. Both mutants MC-1 and SC-E1 are non-motile, lack PFs, and are rod shaped. Mutants MC-1, and one similar to SC-E1 (LC-E1), were previously shown to regain the wild-type phenotype with reintroduction of the respective intact *flaB* or *flgE* gene [13,18,30]. Mutant Δ*W2* has wild-type motility and chemotaxis, and has no discernable altered phenotype [32]. Cells were grown at 34°C in BSK-II liquid medium in 3% carbon dioxide, with the mutants grown in the presence of kanamycin (300 μg/ml) [18]. Approximately 10 ml of *B*. *burgdorferi* cells were grown to late logarithmic phase (7.5 × 10^7^ cells/ml) and centrifuged at ambient temperature for 8 min at 1,800 × *g* and gently resuspended in motility buffer consisting of 136.9 mM NaCl, 8.10 mM Na_2_HPO_4_, 2.7 mM KCl, 1.47 mM KH_2_PO_4_, 2% bovine serum albumin, and 0.1 mM ethylene diamine tetraacetate and adjusted to pH 7.4 [33]. The cell concentration was 7.5 × 10^8^ cells/ml, and held at 0°C in ice. One hour before examination by the AFM, cells were diluted between 5–10 times in 10 mM phosphate buffered saline, pH 7.4, and incubated at ambient temperature.

### Immobilization of spirochetes

Wild-type and mutant cells were immobilized on mica (Ted Pella, Inc.; Redding, CA) that was treated with poly-L-lysine (molecular weight > 30,000; Sigma-Aldrich; St. Louis). The poly-L-lysine coated mica substrates were prepared by depositing 200 μl of poly-L-lysine solution (concentration 0.1 mg/ml) onto freshly cleaved mica, incubating for 1 h, washing with purified water, and drying under a stream of nitrogen. 50 μl of the cell suspensions of either the wild-type or mutants were injected into an AFM fluid cell placed above the mica substrate. After initial injection, the fluid cell was allowed to sit undisturbed for 1 h to ensure sufficient immobilization of the spirochetes.

### AFM analysis

**Fig 2:**
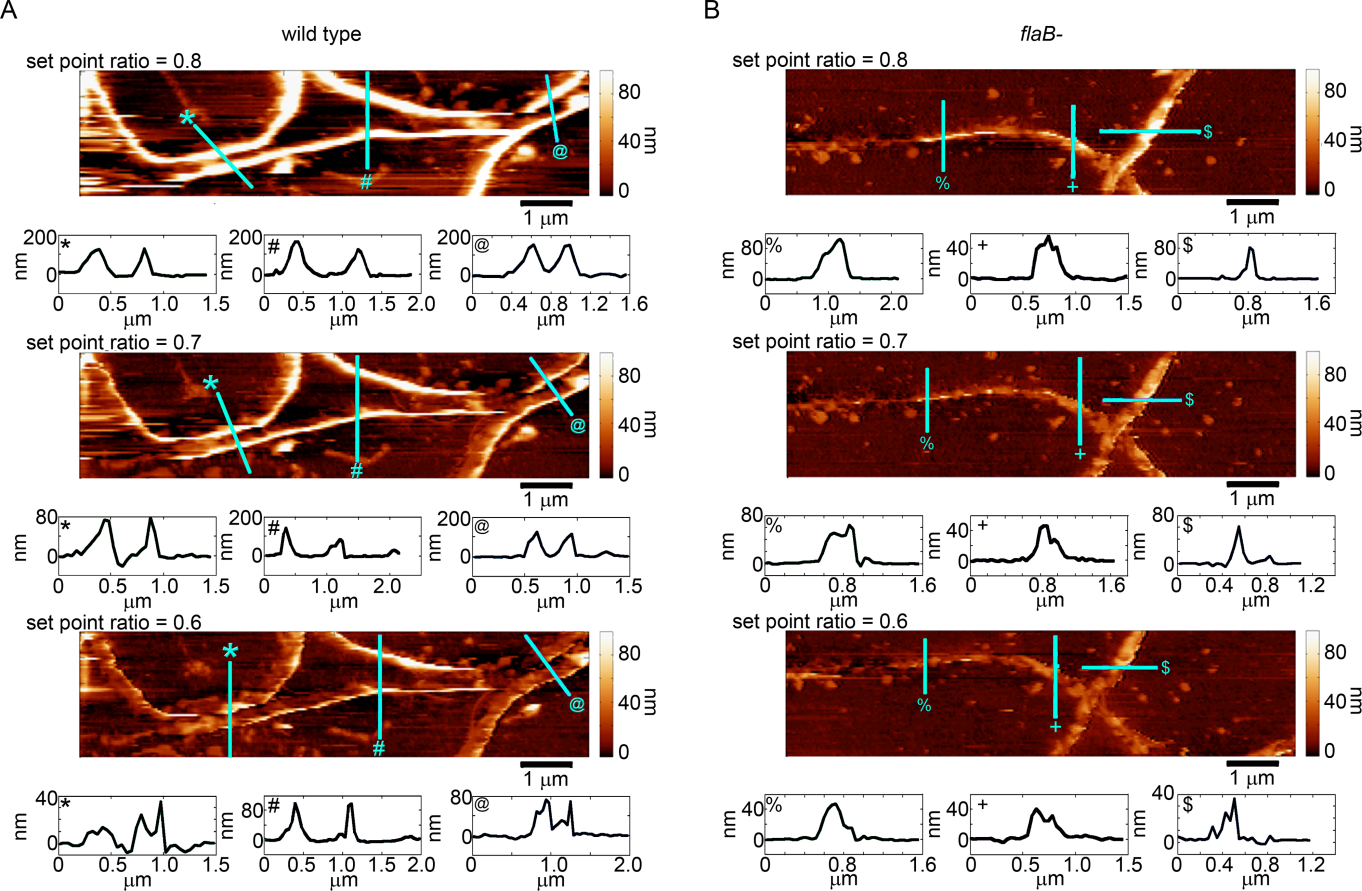
Wild-type spirochetes are more resistant to compression under external stress applied by the AFM imaging process. Sequential tapping mode AFM topography images of (A) wild-type and (B) *flaB* mutant spirochetes of *B*. *burgdorferi* were taken with set point ratios of 0.8, 0.7, and 0.6. The lines indicated by various symbols in the images correspond to the height profiles (indicated by the same symbol) provided directly to the bottom of each image. Note that that the axis limits vary between the different height profiles.

**Fig 3:**
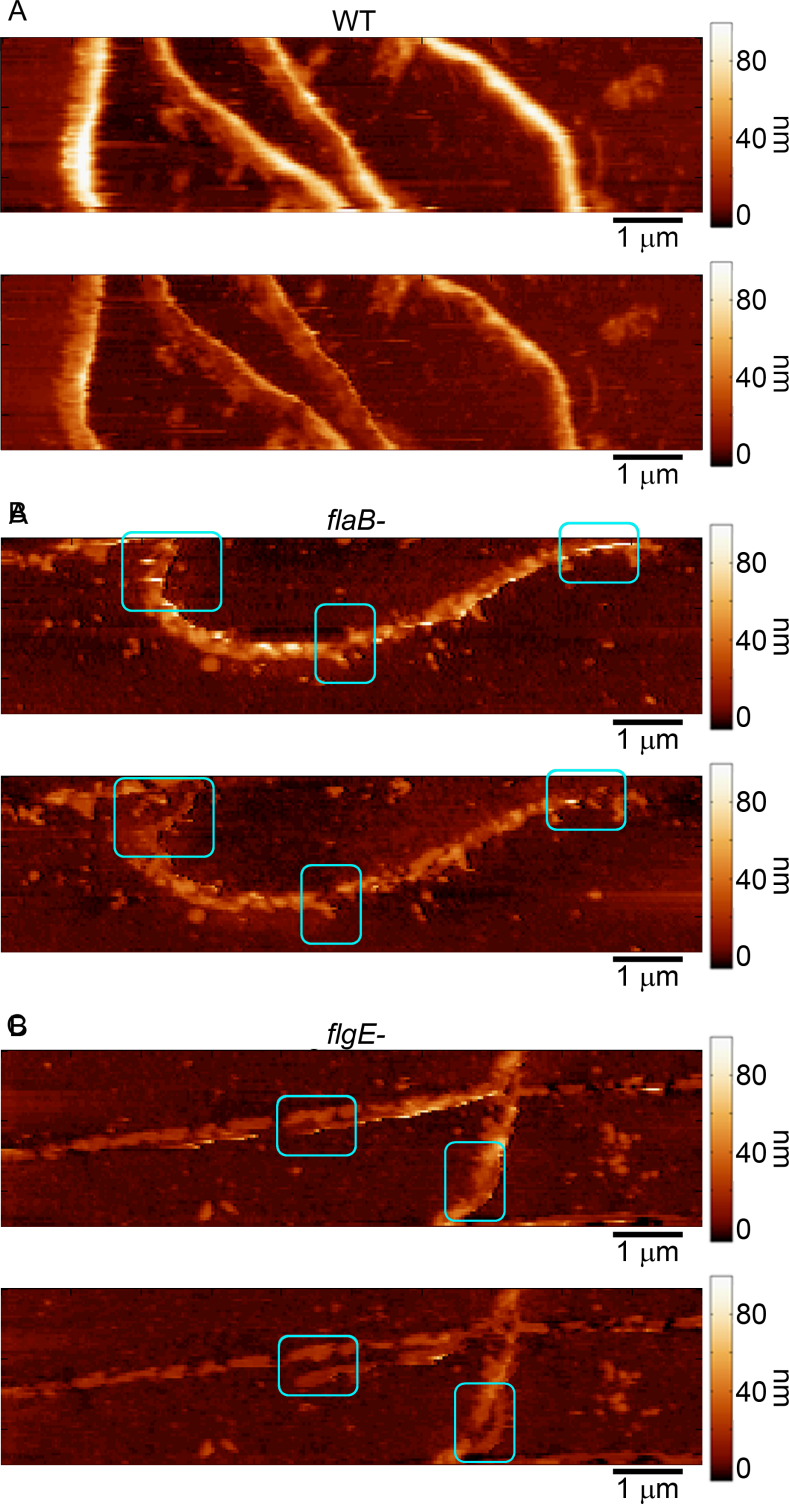
AFM imaging damages mutant spirochetes. Sequential AFM images of (A) wild-type (B) *flaB-* mutant and (C) *flgE-* mutant spirochetes of *B*. *burgdorferi* demonstrating damage associated with increasing imaging forces associated with AFM. Boxes in the images indicate regions (before and after) in which permanent damage to the bacteria was observed.

**Fig 4:**
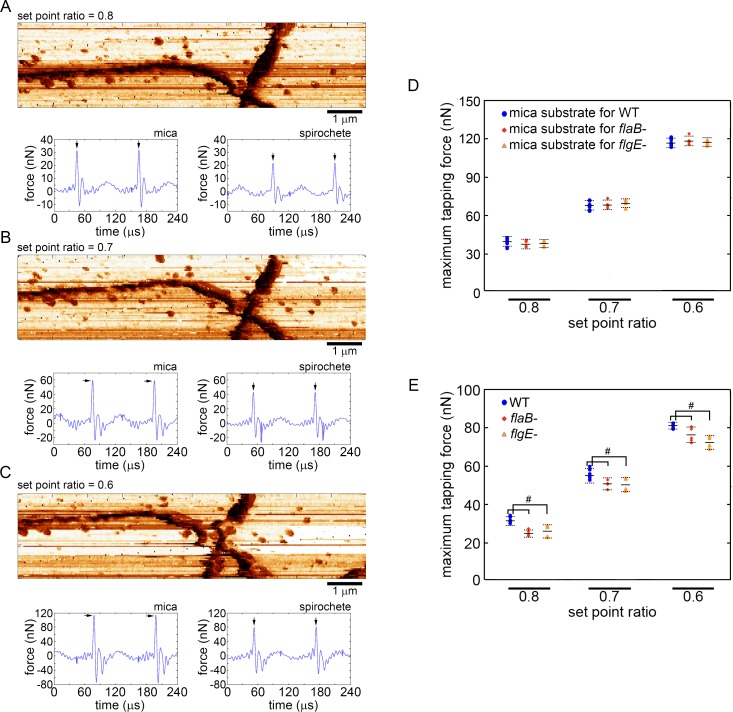
AFM imaging force analysis of wild-type and mutant spirochetes. Reconstructed maximum tapping force images of *B*. *burgdorferi flaB* mutant taken at set point ratios of (A) 0.8, (B) 0.7, and (C) 0.6. The maximum force images correspond to the AFM topography images presented in Fig 2B. Below each image are representative time-resolved tip/sample forces associated with the poly-L-lysine coated mica or spirochete with arrows indicating the maximum force of each tapping event. Two oscillation cycles are shown in each force trajectory. An identical analysis was used for the wild-type (not shown). (D) The average maximum tapping force associated with imaging the mica substrate from experiments with wild-type (n = 10), *flaB* mutant (n = 5), and *flgE* mutant (n = 4) spirochetes as a function of set point ratio are shown. (E) The average maximum tapping force associated with imaging wild-type (n = 10), *flaB* mutant (n = 5), and *flgE* (n = 4) mutant spirochetes as a function of set point ratio are shown. Error bars represent the standard deviation, and # indicates p < 0.05 based on a T-test. The p values for comparing *flaB-* to WT were 0.01, 0.049, and 0.048 for set point ratios of 0.8, 0.7, and 0.6 respectively. The p values for comparing *flgE-* to WT were 0.021, 0.036, and 0.042 for set point ratios of 0.8, 0.7, and 0.6 respectively.

**Fig 5:**
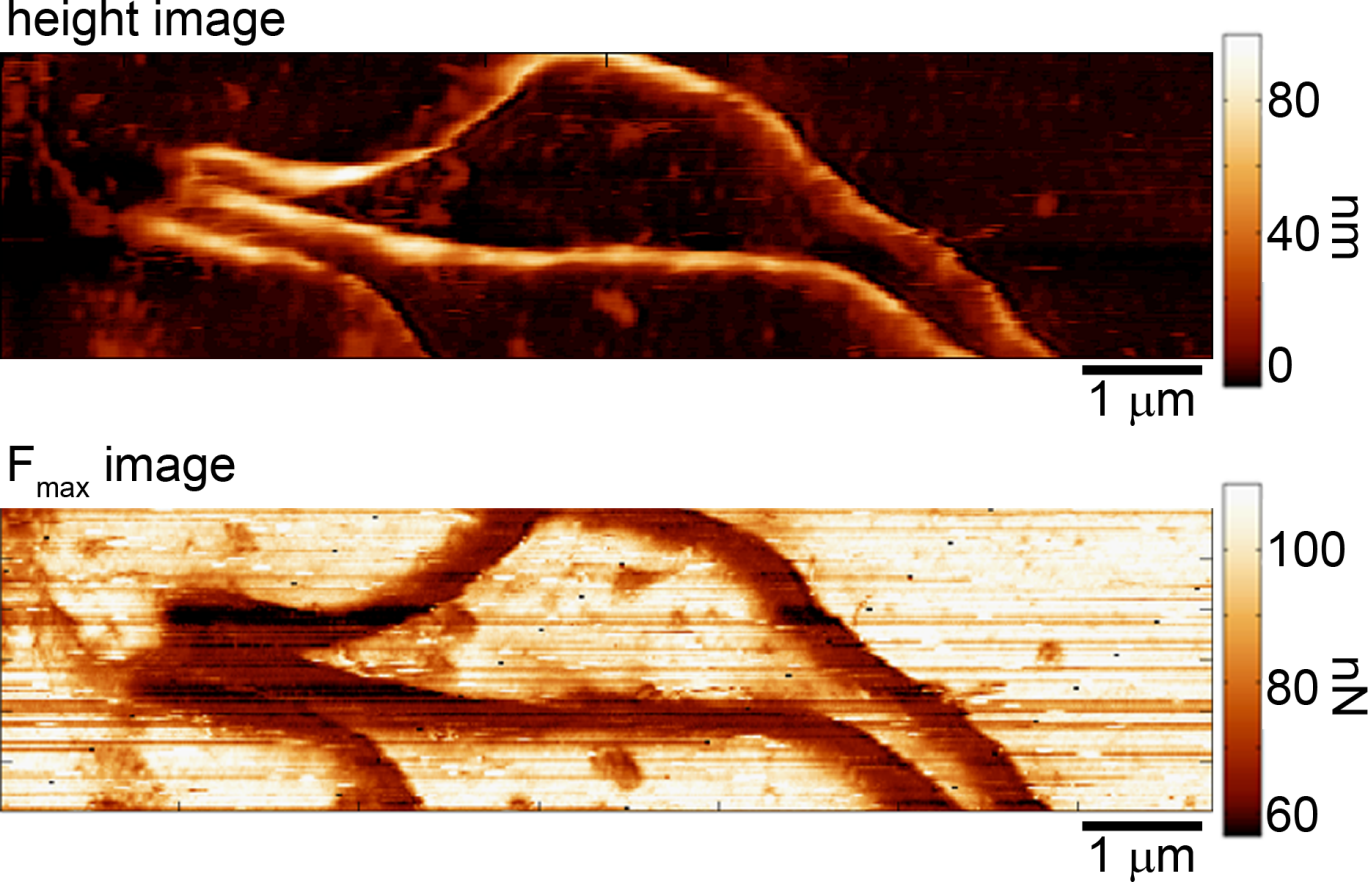
Mechanical properties appear homogenous across spirochete bodies. Corresponding height and reconstructed maximum tapping force images of wild-type *B*. *burgdorferi* taken at a set point ratio of 0.6.

**Fig 6:**
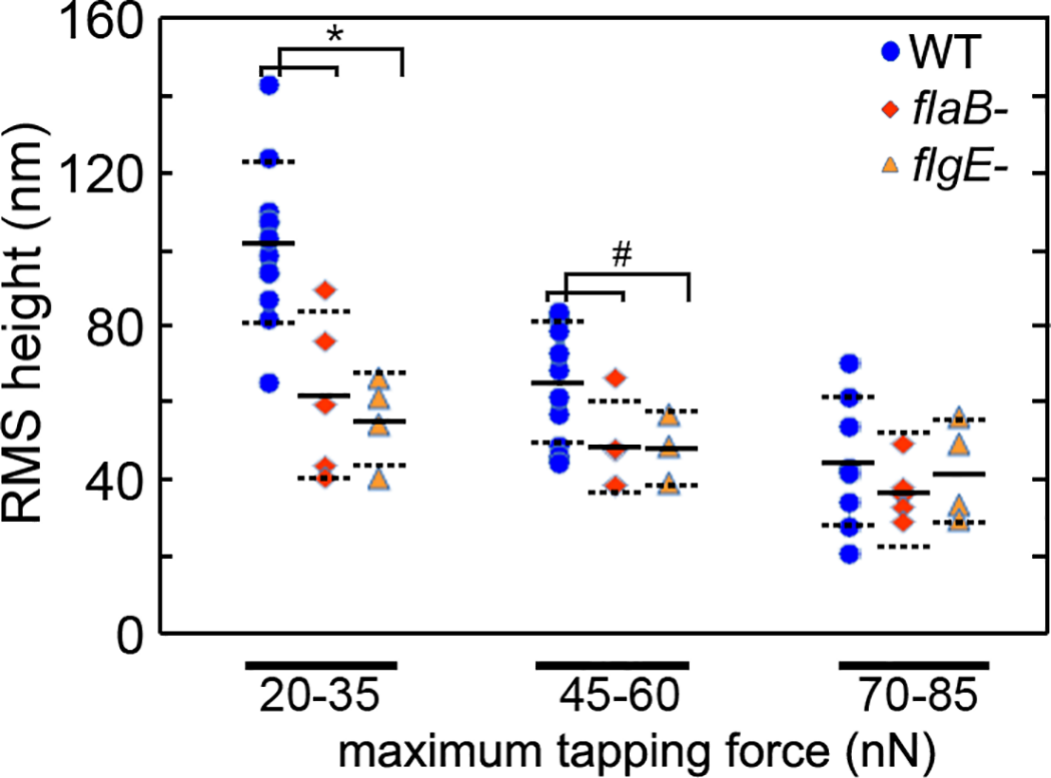
The average height of the spirochetes as a function of applied force. RMS height of wild-type (n = 10), *flaB* mutant (n = 5), and *flgE* (n = 4) mutant spirochetes as a function of the applied maximum tapping force. Average values of RMS height are indicated by the black lines, and the standard deviation is represented by dotted lines.* indicates p < 0.01, and # indicates p < 0.05 based on a T-test.

**Fig 7:**
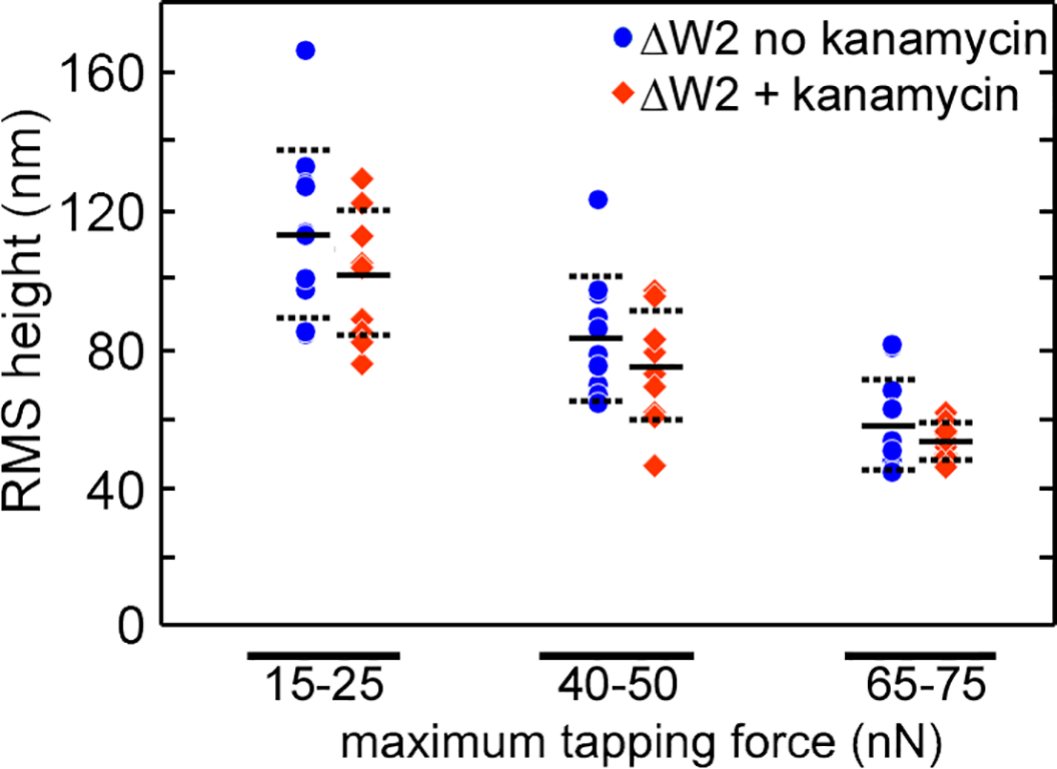
The average height of the mutant Δ*W2* spirochetes as a function of applied force grown in the presence and absence of kanamycin. RMS height of mutant Δ*W2* spirochetes grown in the presence (n = 10) and absence (n = 9) as a function of the applied maximum tapping force. Average values of RMS height are indicated by the black lines, and the standard deviation is represented by dotted lines.

*In situ* AFM experiments were performed with a Nanoscope V MultiMode scanning probe microscope (Veeco, Santa Barbara, CA) equipped with a closed-loop, vertical-engage J-scanner. Images were taken in the tapping mode with V-shaped oxide-sharpened silicon nitride cantilever with a listed spring constant of 0.5 N/m (Veeco). In order to simplify comparisons between experiments, the same cantilever was used to obtain all presented data in Figs 2–6, and this cantilever had a calibrated spring constant of 0.87 N/m (determined by a thermal tuning experiment). In between experiments, the entire fluid cell with the cantilever still inserted was cleaned using a 10% sodium dodecyl sulfate solution followed by separate rinses with nanopure water and ethanol. The fluid cell was then dried with a gentle stream of nitrogen. After each cleaning, the thermal tune was performed again to verify that cantilever was not altered. The scan rate was set at 1.95 Hz with cantilever drive frequencies ranging from ~8–9 kHz. The drive frequency between experiments was varied slightly so that the same drive and free cantilever amplitudes were used for each experiment. The cantilever used to collect the data in Fig 7 was a different cantilever and had a calibrated spring constant of 0.52 N/m.

Scanning probe acceleration microscopy (SPAM) was used to reconstruct the force associated with every tapping event during AFM imaging as previously described [34]. Briefly, cantilever deflection trajectories were simultaneously captured during imaging through an AFM signal access module (Veeco) by using a CompuScope 14-Bit A/D Octopus data acquisition card (Gage Applied Technologies; Lachine, QB, Canada) and custom-written software. Trajectories were captured at 2.5 MS/s and 14-bit resolution. The trajectory of the cantilever was filtered with a Fourier transform based harmonic comb filter that used the first 22 harmonic frequencies of the cantilever. The second derivative of the filtered cantilever trajectory was then taken to obtain tip acceleration, which can be converted to the time-resolved force between the tip and sample by multiplying by the effective mass, *m*_*eff*_, of the cantilever. The *m*_*eff*_ was determined by measuring the resonance frequency, *f*_*res*_, and the spring constant, *k*, of the cantilever as determined by a thermal tuning method [35].

AFM images were analyzed with Matlab equipped with the image processing toolbox (Mathworks, Natick, MA). The root mean square (RMS) height was measured in the following way: (a) Images were imported into Matlab. (b) A flattening algorithm was applied to correct for curvature due to the imaging process. (c) Binary maps of spirochete locations were created from the flattened images by using a height threshold set at 5 nm for all images. (d) The binary maps were used to reference the pixels in the image that were used in calculating the RMS height of the spirochetes.

## Results

To determine the potential role of the PFs on the ability of spirochetes to withstand external stresses, the application of consistent, well-defined external forces was necessary, which can be achieved by careful application of tapping mode AFM in solution. One tapping event occurs during each cantilever oscillation cycle, and the total tip/sample force associated with a single tapping event (*F*_*total*_) can be maintained by using consistent imaging parameters such as spring constant (*k*), drive amplitude, free cantilever amplitude (*A*_o_), and set point ratio (*s*). This can be accomplished by using the same cantilever, which maintains *k*, and using the same drive amplitude to achieve a constant free amplitude. Under such conditions, the applied force to the sample can be maintained and systematically varied by changing the set point ratio (*s = A*_*tapping*_*/A*_*o*_, where *A*_*tapping*_ is the tapping amplitude), with lower ratios resulting in a larger total force applied to the surface during each tapping event [36,37]. The ability to maintain a constant *F*_*total*_ was verified from experiment to experiment by measuring the force associated with imaging the mica substrate, which can be used as an internal reference standard [37,38].

With the ability to systematically apply and maintain different external forces between experiments, we obtained multiple AFM images of immobilized wild-type and mutant spirochetes at a variety of set point ratios imaged in solution (Fig 2). Due to experimental limitations imposed by the SPAM technique used to determine tip/sample forces, images were captured of 10 × 2.5 μm regions of the surface that contained portions of several spirochetes rather than entire cells. The same immobilized spirochetes were analyzed in consecutively taken images as the set point ratio was lowered (from 0.8 to 0.6), systematically increasing the imaging force applied to the spirochetes. The observed height of the spirochetes, for both wild-type and mutants, were considerably smaller than would be expected based on previous EM experiments [13,18], which reported that the thickness of spirochetes with and without PFs are on the order of 300 nm. This smaller than expected height indicates that the spirochetes were being compressed, even at a set point ratio of 0.8 that is associated with the smallest imaging force applied in this study. Furthermore, the observed height of the wild-type and mutant spirochetes became noticeably smaller due to increased compression and radial deformation caused by the elevated imaging forces associated with decreasing the set point ratio. These induced changes can be clearly seen in the height traces provided below each image in Fig 2. We found that as the applied force became larger, the observed height of the spirochete above the surface decreased. However, the spirochetes lacking PFs appeared to be compressed to a greater extent (Fig 2B) compared to their wild-type counterparts (Fig 2A).

The process of varying the imaging force could consistently be repeated on wild-type spirochetes without typically inducing permanent morphological changes, indicating that increased compression did not damage the cell. Permanent damage of the cell was observed in 10% of the wild-type spirochetes (n = 20) in multiple experiments with two different cantilevers. However, mutant spirochetes more often displayed small regions of permanent damage (areas where the cell body appeared to break or splinter) associated with the highest applied imaging force (Fig 3). While permanent damage was not apparent for every individual mutant spirochete imaged, it was frequently observed for both the *flaB-* and *flgE-* mutant spirochetes (53% of the mutant spirochetes imaged, n = 19) in several experiments, suggesting that PFs may function to help the cell maintain its integrity against externally applied forces.

While *F*_*total*_ remains constant for any given set of imaging parameters, several numerical models and experiments have demonstrated that the maximum tapping force (*F*_*max*_), defined as the peak or largest positive force associated with a tapping event, increases with a power law dependence as sample elastic modulus increases [36,37,39,40]. As a result, *F*_*max*_ can be used to obtain surface maps with contrast indicative of variations in elastic modulus of the sample surface (Fig 4A–4C). The time-resolved tip/sample force associated with every tapping event can be reconstructed from the cantilever deflection signal using SPAM [34,41]. As can be seen from the time-resolved force trajectories, each tapping event results in a pulsed force interaction, and the reconstructed tapping forces confirmed that the total tapping force applied to the sample increased with decreasing set point ratio and that similar *F*_*total*_ was applied between experiments. The magnitude of *F*_*max*_ was significantly larger for tapping events occurring on the exposed poly-L-lysine coated mica compared with those associated with wild-type or mutant spirochetes cell bodies, which is consistent with spirochetes having a lower elastic modulus than the mica substrate. By using the same cantilever and maintaining imaging parameters (drive amplitude, free amplitude, and set-point ratio), the relative rigidity associated with the wild-type and mutant spirochetes could be indirectly assessed by comparing the magnitude of *F*_*max*_. At any given set point ratio, *F*_*max*_ associated with wild-type spirochetes was statistically larger compared with the *F*_*max*_ associated with both *flaB-* and *flgE-* mutants (Fig 4E). Because *F*_*max*_ is associated with a larger elastic modulus, our results suggest that the PFs provide structural stability to spirochetes and provide resistance to compression. The maximum tapping force associated with imaging *flaB-* and *flgE-* mutants were statistically indistinguishable from each other. As an internal control, the maximum tapping force associated with imaging the poly-L-lysine coated mica was not significantly different when compared across all samples (Fig 4D). This result verifies that we were able to reproducibly apply the same force from experiment to experiment, allowing for direct comparison between wild-type and mutant spirochetes. However, it should be noted that while the SPAM technique provides consistent, reproducible measurements of tip/sample forces, it overestimates the actual magnitude of the applied imaging force [42]. For this reason, quantification of the actual elastic modulus of the different spirochetes was not achieved.

Conceivably, there could be stiffness variations along the body of the wild-type spirochete; that is, cell stiffness as measured by the probe could be a function of the orientation of the PF ribbons (e.g. if the ribbon was directly under the probe vs it being at 90°). However, contrast within the cell body in F_max_ images associated with wild-type spirochetes was not observed (Fig 5). This lack of force contrast within the cell body may be due to the compression associated with the imaging process. Specifically, the height data confirms that the spirochetes are being compressed under the applied imaging force. This cell compression by the probe results in an increase in the contact area between the probe and spirochete. Lateral spatial resolution in AFM is highly dependent on this contact area, limiting our ability to potentially see stiffness variation along the spirochete body.

We quantitatively evaluated the average height of the spirochetes as a function of applied force (Fig 6). The RMS height of regions of the image containing spirochetes was used as a measure of average spirochete height and compared based on *F*_*max*_. We used the RMS height rather than comparing height measurements at discrete points, as the height along the cell body can vary. Thus, height measurements are highly dependent on where along the cell the measurement was taken. However, the RMS height measurement reflected changes over the entire cell. Rather than performing the RMS analysis over entire images, only regions of the image containing spirochetes were used in this analysis to prevent bias associated with varying surface coverage. Using geometrical models, cylindrical bodies lying flat on a surface without any deformation would have an RMS height of ~90% of the diameter of the cylinder. Based on this, it would be expected that the observed RMS height of a spirochete with a diameter of ~300 nm with no deformation would be ~270 nm. However, the average RMS heights were significantly smaller for all samples, even at the lowest applied force, confirming that the spirochetes are undergoing considerable compression associated with the imaging process. At applied maximum force of 20–35 nN (set point ratio 0.8), the average RMS height of wild-type spirochetes was 102.2 ± 19.8 nm. This observed RMS height was statistically larger compared with the RMS height of *flaB-* (62.6 ± 20.9 nm, p = 0.01) and *flgE-* (56.3 ± 11.2 nm, p = 0.005) mutant spirochetes imaged in this force range. At applied maximum force of 45–60 nN (set point ratio 0.7), the wild-type spirochetes were further compressed with an average RMS height 65.4 ± 14.9 nm. While the compression of *flaB-* (48.7 ± 11.4 nm) and *flgE-* (49.0 ± 8.8 nm) mutant spirochetes also increased (smaller RMS height), the wild-type spirochetes still underwent significantly less compression (p = 0.037 and p = 0.049 respectively). As the applied maximum force exceeded 70 nN (set point ratio 0.6), a statistically significant RMS height difference between the wild-type (45.1 ± 15.9 nm) and mutant spirochetes (37.8 ± 14.6 nm and 42.7 ± 12.7 nm for *flaB-* and *flgE-* respectively) was no longer observed. For any given applied force, the RMS height of the *flaB-* and *flgE-* spirochetes were statistically indistinguishable from each other. Collectively, these observations suggest that the presence of PFs provide the spirochete with the ability to withstand compression associated with external stress.

We were concerned that kanamycin added to the growth medium of *flaB-* and *flgE-* cells altered the cellular structure of these mutants. Thus, even though the *aphI* cassette encodes a kinase that phosphorylates and inactivates kanamycin, perhaps growth in kanamycin and not the absence PFs is responsible for the susceptibility to compression compared to the wild-type. To test for this possibility, we analyzed mutant Δ*W2* of *B*. *burgdorferi* that has an *aph1* insertion deletion mutation within the chromosomal *cheW2* gene; this mutant has the wild-type phenotype with respect to motility and chemotaxis [32]. We subcultured mutant Δ*W2* nine times with kanamycin, and also nine times without kanamycin, and their susceptibility to compression was determined (Fig 7). Note, this experiment was performed with a different cantilever than those presented in Figs 2–6, and as a result, the tapping forces generated were slightly smaller in magnitude. This resulted in moderately larger values of RMS heights observed. Still, the results on the Δ*W2* spirochetes, which have PFs, are comparable to those previously presented for wild-type spirochetes. That is, the RMS height of both the Δ*W2* and wild-type spirochetes decreased with increasing applied tapping force. In addition, no significant difference in the RMS height of spirochetes containing PFs was observed in the absence or presence of kanamycin (p = 0.16, 0.24, and 0.16 for the respectively applied forces), confirming that the presence of kanamycin was not responsible for the observed susceptibility to compression.

## Discussion

It is well established that the PFs play an important cytoskeletal role in determining the morphology of B. *burgdorferi* [1,3,4,7,10,25]. Bacterial cells and flagella are elastic materials, deforming under applied stress and reverting back to their original shape upon removal of a stress. The flat-wave morphology of *B*. *burgdorferi* arises from the interaction and forces between the helical PFs and the rod-shaped protoplasmic cell cylinder [10,18,24,25]. We have shown that PFs also play a role in withstanding externally applied stresses and enhancing cellular integrity. This resistance to applied stress is likely due to the PFs, which form tight filament-ribbons that wind around the protoplasmic cell cylinder [7,14–16], dissipating energy applied to the exterior of the cell body. The ability of fibrous cytoskeletal structures to dissipate energy is a common phenomenon in other cellular systems [43,44]. While a potential caveat is that removal of the PFs caused a separate cellular response that accounts for the altered ability to withstand external stress, we feel this is unlikely due to two reasons. First, it is clear, for example, that PFs are stiffer than the cell body by the influence that they have on overall spirochete shape [10]. Second, the PFs were removed in two different models via distinct mutations, and this resulted in similar observations associated with the application of external stress to these mutant cells. Gene transfer experiments indicate that each of the mutants only suffered one mutation [13,18,30]. Thus, secondary mutations in the mutants were not responsible for the lack of PFs and flat-wave morphology.

The forces involved with cell shape and motility in *B*. *burgdorferi* are complex and are not completely understood. Specifically, the rod-shaped cell cylinder is converted to a flat wave by mechanical forces exerted by the PFs [10]. Likewise, the shape of the PFs is altered by this interaction [10]. Purified PFs are left handed, are polymorphic, and have a helix pitch of either 1.4 μm or 2.0 μm [10,45]. However, *in situ* the helix pitch of the PFs is altered to 2. 83 μm [20]. Thus, the flat wave morphology is the consequence of the ribbons exerting force on the rod shaped cell cylinder, and the cell cylinder exerting an opposite and equal force on the PFs [10]. The rod-shaped cell cylinder, whose shape is determined by the peptidoglycan, is quite flexible. For example, swimming cells can have a distorted morphology and even bend in the central part of the cell during swimming (supplemental movie 3 of rev 7) [46]. As noted, the flagellar hook in *B*. *burgdorferi* is unique, as its FlgE proteins are covalently cross-linked to one another [7,12,13]. In contrast, in other bacteria, the hook proteins are less tightly held together by electrostatic and hydrophobic bonds. It is not clear why spirochete hook proteins are cross-linked and presumably stronger, but at least for one species of spirochete, *T*. *denticola*, this cross-linking is essential for translational motility [12]. Whatever the reason why the hook structure is cross-linked, it illustrates that we know very little concerning the intricate details relative the forces that govern shape, stability, and motility of *B*. *burgdorferi*. Here we show that cells lacking PFs are more susceptible to external stress, and our results suggest that the ribbon stabilizes the cell cylinder by effectively functioning as a wide belt or girdle.

There are obvious advantages of PFs, such as efficient motility in highly viscous gel-like environments, attracted to and attaching to tissues, moving along tissue surfaces, and organ penetration [1,3,4,7,19,21,28,29,47–49]. The ability demonstrated here to dissipate energy associated with external stresses represents another advantage associated with PFs. The enhanced structural integrity conferred by PFs may allow spirochetes to better survive in high pressure environments, such as the bloodstream and when attached to endothelial cells. Along these lines, PF deficient *flaB* mutants derived from virulent *B*. *burgdorferi* have recently been shown to be less able to propagate both in ticks and in mice [3,4,19]. We propose that part of the reason for their lack of propagation is their more susceptibility to mechanical stresses within these environments. Cell wall stiffness varies considerably among different bacterial species, and large variations in cell cylinder stiffness are also apparent between different spirochete species [10,22]. While the individual components of spirochetes are elastic, large enough applied stresses could potentially induce mechanical failure. Our studies indicate that the absence of PFs reduces the overall elastic modulus of the cell with concomitant reduction in its ability to avoid mechanically-induced damage. Furthermore, there is a limit to how much a spirochete can be compressed. As the mutant spirochetes lacking PFs are more easily compressed, the cells reach this maximum compression at smaller applied forces, making them more susceptible to permanent damage. The ability to prevent irreversible damage due to compression by an external force may play a role in the ability of *B*. *burgdorferi* to penetrate gelatin mimics with pore sizes smaller than the bacterial diameter [48].

As the stiffness of a group of PFs increases with the number of filaments, increasing or decreasing the number of PFs appears to be another factor that influences overall cell shape and cell stiffness. Along these lines, modulation of the CsrA protein markedly influences FlaB synthesis and thus both the length and number of the PFs, and concomitantly, cell shape [16]. Specifically, mutant cells that overproduce CsrA are rod-shaped with fewer and shorter PFs, and cells that under produce CsrA have longer PFs with a smaller cellular wave length than the wild-type. The PFs under this low CsrA condition wind around the protoplasmic cell cylinder tighter with a smaller helix pitch than the wild-type. Based on this observation, we speculate that spirochetes could conceivably adjust the number of PFs in response to external pressure associated with their environment to not only optimize motility as suggested by Dombrowski et al. [10], but as suggested here, compensate for physical stresses and maintain cellular integrity. Along these lines, *Vibrio parahaemolyticus* can sense the viscosity of the environment, or whether it is near a surface, and is able to markedly change its flagellar configuration (lateral vs polar) and its cell length [50].
